# Stent Graft Placement for the Treatment of Hepatic Artery Pseudoaneurysms: A Single-Center Experience

**DOI:** 10.7759/cureus.100326

**Published:** 2025-12-29

**Authors:** Stephanie Woo, Sun Yu Lam, Lik Fai Cheng

**Affiliations:** 1 Department of Diagnostic and Interventional Radiology, Princess Margaret Hospital, Kowloon, HKG

**Keywords:** coil, embolization, hepatic artery pseudoaneurysm, stent graft, whipple's operation

## Abstract

Introduction: Hepatic artery pseudoaneurysms (HAPs) represent a rare but serious complication after major pancreatic, hepatic, or gastric surgery. Apart from surgical repair, endovascular treatment such as stent graft placement or coil embolization is a less invasive alternative in these patients. Among the endovascular options, treatment of pseudoaneurysms by stent graft placement has the advantage of preserving hepatic arterial perfusion, which reduces the risk of hepatic ischaemia and major postoperative complications in these patients. The purpose of our study was to evaluate the technical feasibility, efficacy, procedural complications, and outcomes, including graft patency in HAPs treated with a stent graft.

Methods: A retrospective analysis of patients who had undergone endovascular treatment of HAPs with stent grafts between January 2021 and January 2024 was conducted. Patient characteristics, including age, sex, surgical history, and type of cancer on final pathology, were recorded. Angiographic data on target vessel, material used, and technical success, defined as the exclusion of the pseudoaneurysm by means of a stent graft with sufficient control of bleeding, any major complications, and follow-up data, were collected.

Results: A total of seven patients were included, five of them were male patients (71.4%), while two of them were female patients (28.6%). Mean age was 70.8 (range: 59-75). All of our patients were postoperative patients who developed HAPs, likely secondary to anastomotic dehiscence. Stent graft deployment was technically successful in six of the patients (85.7%). One case was unsuccessful due to vascular tortuosity, leading to a dislodged stent graft, which was subsequently repositioned and deployed at a branch of the splenic artery. The HAP was embolized with coils. No major procedural complication was recorded. Complete exclusion of the pseudoaneurysm while preserving patency of the hepatic artery was achieved in all of the patients in which stent graft deployment was technically successful. A follow-up enhanced computed tomography scan was performed at an average of 3.3 months (range: one to eight months), which showed the disappearance of HAP and patency of the stent without in-stent stenosis. In patients who had successful deployment of the stent, six-month all-cause mortality was 42.7% (three out of seven). Two of the patients died of chest infection, while the other patient died of uncontrolled sepsis. None of the patients died of bleeding due to liver failure.

Conclusion: Endovascular treatment of HAPs using stent grafts is technically feasible and has the potential benefit of maintaining hepatic artery blood flow, and could be considered as an alternative to surgery and other endovascular treatments.

## Introduction

Stent graft placement offers a viable treatment for visceral arterial injuries, with the key benefit of maintaining arterial flow, unlike embolization [1,2]. While hepatic artery injuries and bleeding from the gastroduodenal artery (GDA) stump are rare, they can lead to severe complications following pancreaticoduodenectomy and other abdominal oncologic surgeries [2]. Endovascular coil embolization is one approach to manage such bleeding, but it often necessitates blocking the parent hepatic artery, which carries risks of hepatic ischemia or infarction [3]. A treatment alternative is hepatic artery stent graft placement, which aims to maintain hepatic arterial perfusion [4] and preserve future options for locoregional transarterial therapies. This retrospective cohort study assesses the technical feasibility, safety, and patency rates of hepatic arterial stent grafts used to treat hepatic artery pseudoaneurysms (HAPs).

This article was previously presented as a meeting abstract at the European Conference on Interventional Oncology 2024 and at the 18th Annual Scientific Meeting of the Asia Pacific Society of Cardiovascular and Interventional Radiology.

## Materials and methods

This retrospective study was performed at a regional hepatobiliary tertiary hospital in Princess Margaret Hospital, Kowloon, Hong Kong. Prior to the commencement of this retrospective study, approval from the Hospital Authority Central Institutional Review Board (Central IRB) was obtained. All patients treated with stent graft placement for HAPs from a variety of clinical conditions from January 2021 to January 2024 were included. Patients with incomplete records and stent grafts placed for causes other than HAPs were excluded.

The electronic patient record and the Radiology Information System of the institutions were utilized to collect the clinical data of the included patients. This study included a review and assessment of the hospital charts, electronic medical records, and images of patients, focusing on parameters including patient sex, age, diagnosis, type of surgery undergone, embolization technique, adverse events, results of follow-up imaging, and recurrence of bleeding episodes.

Follow-up imaging was reviewed to evaluate stent graft patency. Imaging studies were independently reviewed in a blinded manner by two radiologists (SC, SY, eight years and 15 years of radiologic experience, respectively). In cases of discordance, the studies were independently reviewed by an additional radiologist (LF, 25 years of radiologic experience).

Technical success rate and clinical success rate with 95% confidence intervals (CIs) were calculated. Technical success [1] was defined as the exclusion of the pseudoaneurysm by means of a stent graft with sufficient control of bleeding. Clinical success was defined as resolution of bleeding from the target site within 30 days with no need for additional intervention. The clinical success rate was calculated by subtracting the patients with rebleeding from the target site within 30 days from the total number of reported successful stent-graft procedures [5].

The maintenance of a regular blood flow to the liver arteries on the final angiogram, as well as on follow-up imaging, was evaluated. The follow-up CT performed for all patients had a uniform protocol with dedicated arterial phase studies for optimal evaluation of the stent graft patency.

The electronic medical records were examined to assess whether patients received anticoagulation or antiplatelet therapy following stent graft placement. Complications occurring within 30 days of the procedure that were directly linked to the stent graft placement were considered. These adverse events were categorized and reported in accordance with the adverse event grading standards set by the Society of Interventional Radiology [6].

## Results

Patient population

A total of seven patients were included in this study, comprising five males and two females, with a mean age of 70.8 years (range: 59-75). Patient demographics, cancer diagnosis, and procedural details are listed in Tables 1-2.

**Table 1:** Patient demographics

Characteristic	Value
Mean age	70.8 (range: 59-75)
Gender	
Male	5
Female	2
Oncologic diagnosis	
Pancreatic	3
Ampulla of Vater	2
Others	2
Type of surgery undergone	
Whipple’s operation	5
Central pancreatectomy	1
Radical gastrectomy	1
Follow-up imaging modality	
CT	7
Mean follow-up timing of CT	3.3 months (range: 1-8 months)

**Table 2:** Procedural details

Characteristic	Value
Access: femoral	7
Stent graft number	
1	4
2	3
Stent graft type	
PK Papyrus coronary	4
BeGraft	3
Target vessel	
Common hepatic artery	5
Right hepatic artery	2
Additional embolic agent	
Coils	3

**Figure 1:**
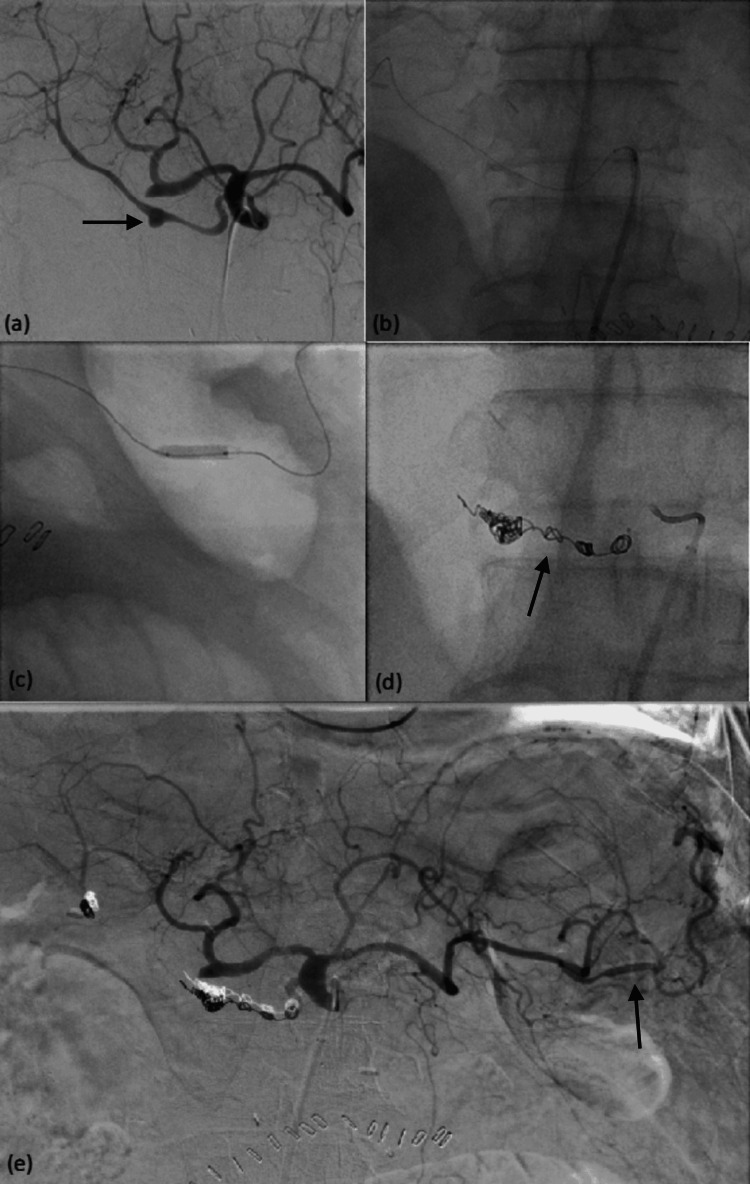
A 74-year-old male patient who had undergone Whipple’s operation and had known pancreatojejunostomy dehiscence presented with shock. Angiography shows a small pseudoaneurysm (black arrow) at the proximal right hepatic artery (a). Stent graft insertion was initially planned; however, the stent graft dislodged at the origin of the right hepatic artery due to vascular tortuosity (b). The dislodged stent graft was then redirected and deployed in the inferior division of the splenic artery (c). The pseudoaneurysm was treated with coils (black arrow, d). Post-embolization angiography shows obliteration of the pseudoaneurysm, with patency of the inferior division of the splenic artery (black arrow, e).

**Figure 2:**
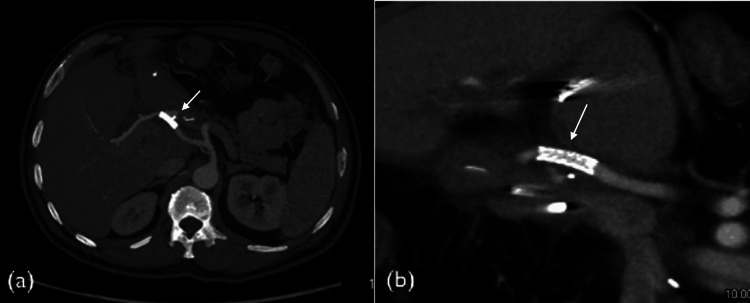
Early follow-up CT scan in a patient with a common hepatic artery stent graft. Maximum intensity projection images in the axial (a) and sagittal (b) planes obtained two months after implantation of a 5 mm × 22 mm BeGraft stent (white arrows) demonstrate patency of the stent.

Stent graft deployment was technically successful in six patients (85.7%, 95% CI = 59.8-100%). Stent deployment was not possible in one of the cases, resulting in the dislodgement of the stent because of the tortuous anatomy of the common hepatic artery. The stent graft was able to be directed to the splenic artery for deployment, and the hepatic artery was trapped using coils (Figure 1). In all cases where deployment was successful, complete exclusion of the pseudoaneurysm was achieved while preserving hepatic artery patency. Clinical success was achieved in all patients (100%, 95% CI = 100-100%). Follow-up enhanced computed tomography angiography was performed at an average of 3.3 months (range: one to eight months) post-procedure, showing the disappearance of the HAP and maintenance of stent patency without evidence of in-stent stenosis (Figure 2). No major procedural complications were observed. The six-month all-cause mortality was 42.7% (three out of seven), with two deaths attributed to chest infections and one to uncontrolled sepsis. Importantly, no deaths were related to bleeding or liver failure.

Procedural techniques

**Figure 3:**
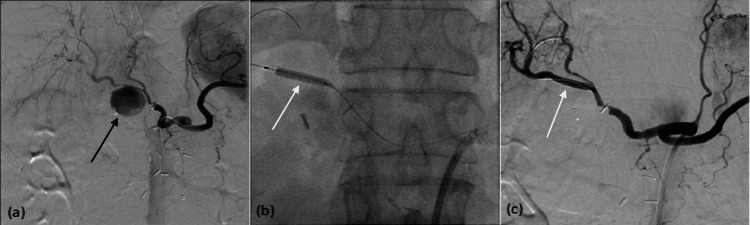
A 75-year-old patient who underwent radical pancreaticoduodenectomy for pancreatic cancer. Digital subtraction angiography demonstrates a large pseudoaneurysm (black arrow) of the right hepatic artery 15 days after surgery (a). A 3 mm × 20 mm PK Papyrus stent graft (white arrows) was placed across the pseudoaneurysm neck to exclude it, shown during (b) and after deployment (c).

**Figure 4:**
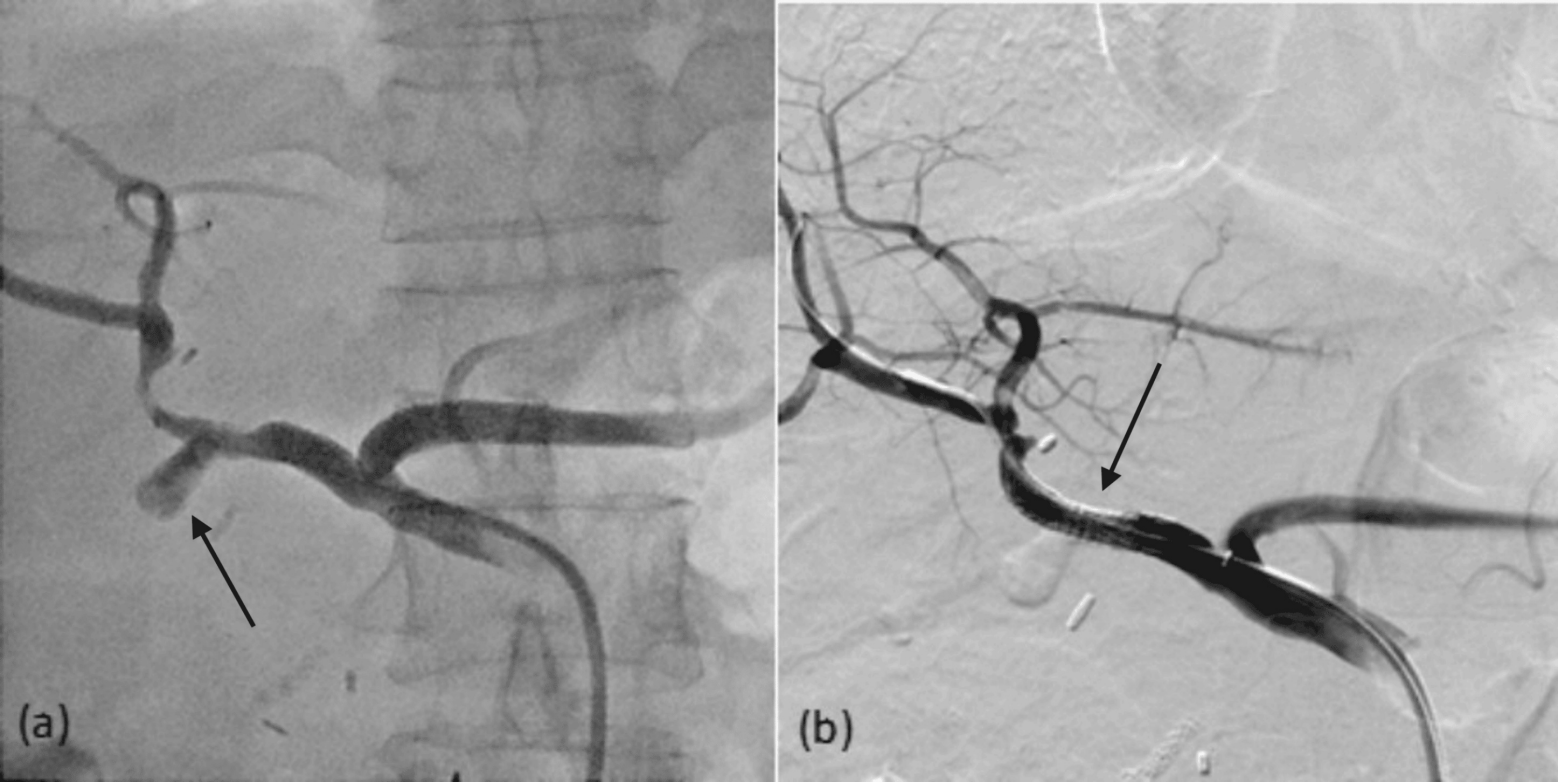
A 72-year-old male patient who underwent central pancreatectomy and presented with hemodynamic instability. Angiography shows a common hepatic artery pseudoaneurysm (black arrow, a). After stent graft placement, the pseudoaneurysm is obliterated (black arrow, b) with preserved flow in the common hepatic artery.

Treatment was performed under local anesthesia in all patients. Transfemoral access was used in all patients, with a minimum size of the introducer sheath of 4F. Then, a 4F diagnostic catheter was advanced into the celiac artery using either a Cobra 1 catheter (Cook Medical, Bloomington, IN, USA) or a Shepherd Hook catheter (Merit Medical Systems, South Jordan, UT, USA). The target vessel was the common hepatic artery in five out of seven patients and the right hepatic artery in two out of seven patients (Figures 3, 4).

**Figure 5:**
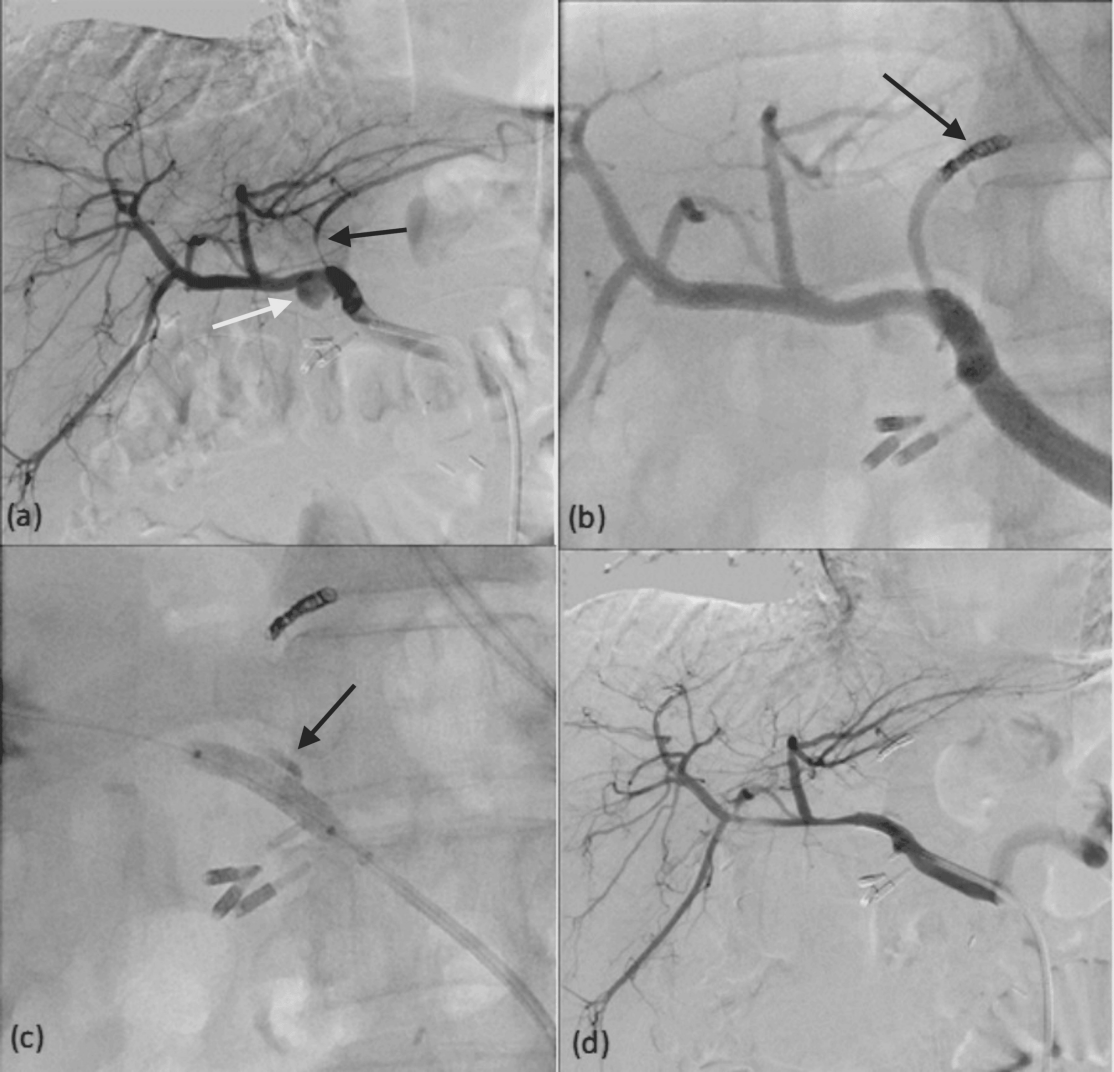
A 59-year-old male patient who had undergone Whipple’s operation and presented with hemorrhagic shock. Angiography shows a pseudoaneurysm (white arrow, a) arising from the common hepatic artery near the origin of the left gastric artery (black arrow, a). Coil embolization was performed in the left gastric artery (black arrow, b). A 5 mm × 22 mm BeGraft stent graft was then deployed across the pseudoaneurysm (black arrow, c). Post-procedural angiography demonstrates obliteration of the pseudoaneurysm (d).

Additional coil embolization was performed in two patients for arteries arising from the intended zone of placement of the stent graft, for prevention of endoleak, including coil embolization of the GDA and left gastric artery (Figure 5).

The target vessel's diameter was determined using either pre-procedural cross-sectional images or intra-procedural angiography images. The stent graft was sized 1 mm larger than the measured vessel, as per institutional practice. A long vascular sheath - either a Destination guiding sheath (Terumo Medical Corporation, Tokyo, Japan) or an Ocor steerable guiding sheath (Integer Holdings Corporation, Plano, TX, USA) - was then positioned at the origin of the celiac artery to provide adequate support. The pseudoaneurysm was crossed using a microguidewire compatible with the stent graft delivery system, either a Transcend or Asahi Chikai guidewire (Asahi Intecc Medical, Aichi, Japan) or a Synchro Support guidewire (Stryker, Kalamazoo, MI, USA). The stent graft was then inserted through the sheath and deployed across the pseudoaneurysm. The most commonly used stent grafts in our center were PK Papyrus (Biotronik, Berlin, Germany) and BeGraft (Bentley InnoMed, Hechingen, Germany), both of which are balloon-expandable stents. Following stent placement, any additional balloon dilatation or extension of the stent graft was based on the effectiveness of pseudoaneurysm exclusion observed on post-deployment angiography.

Weight-based bolus heparin (60 u/kg) was administered for all patients during the intervention after the stent graft had been appropriately placed. Oral administration of antiplatelet medications would be initiated after the intervention, depending on the clinical scenario, and after discussion with the referring clinical team, determined on a case-by-case basis.

## Discussion

Stent graft implantation proved to be an effective method for treating pseudoaneurysms of the hepatic artery, achieving a technical success rate of 86% in excluding the pseudoaneurysms in our series. These findings align with current literature, which reports a technical success rate exceeding 85% [2,7-9]. In the case of failed stent graft deployment, the patient had a very tortuous anatomy of the common hepatic artery, resulting in the dislodgement of the stent. The stent graft was able to be directed to the splenic artery for deployment, and the hepatic artery was trapped using coils. To minimize such occurrences, it is important to have a good patient selection with favorable anatomy and to avoid using excessive force during the advancement of the stent graft over the wire. Additionally, appropriate interventional tools, for example, long sheaths and stiff wires, would also help to provide additional support for the system, which helps minimize the possibility of failed stent graft deployment.

Clinical success was achieved in 100% of the patients where stent graft placement was technically successful, which is in line with current literature, reporting a clinical success rate of 91-93% [5,10,11]. Despite a high clinical success rate, the six-month all-cause mortality was 42.7%, with two deaths due to chest infection and one death due to uncontrolled sepsis. This is likely due to the patients who required stent graft placement were all oncologic patients with prolonged hospital stays, which increases their risk of early mortality. None of the patients died of hepatic arterial bleeding or liver failure.

In our series, 100% of the cases with successful stent graft placements maintained hepatic arterial flow during short-term follow-up. In terms of patency rates, the reported primary patency rates differ widely across the literature depending on the duration and method of follow-up. Hassold et al. [12] reported stent patency rates of 84% at 30 days after implantation, while Bellemann et al. [13] reported patency rates of 89% at four months after implantation.

There were no major complications in this series. Possible adverse events related to hepatic arterial stent grafts that are reported in the literature include rapid stent graft occlusion or arterial dissection during placement, leading to ischemic injury or infarction [1,12]. This risk is also present with endovascular coil embolization of the hepatic artery, which is an alternative treatment option.

The anticoagulant and antiplatelet therapy protocols implemented after stent graft implantation also vary widely across the literature, with no established guidelines. In our centre, the choice of antiplatelet or anticoagulation regimen was primarily determined by the operator's judgment, taking into account the patient's bleeding risk.

The limitations of this study include its retrospective and single-centre design. The low incidence of HAPs restricts the ability to gather a larger and more diverse patient population, leading to a small sample size. Additionally, the lack of standardized follow-up imaging studies due to differing referral protocols limits our evaluation of long-term patency rates.

## Conclusions

To conclude, stent graft placement for HAPs demonstrates significant safety and effectiveness as a treatment strategy, as evidenced by high short-term patency rates. This approach offers a crucial advantage over traditional coiling methods, particularly in its ability to preserve hepatic arterial flow, which is vital for preventing liver ischemia, maintaining liver function, especially in oncological patients who have already undergone major surgery.

Future studies with larger cohorts and extended follow-up periods are essential to validate the longevity of benefits associated with this approach and to fully assess its role in clinical practice. While stent graft placement appears to be a promising alternative to traditional methods for treating HAPs, generating robust long-term data will ultimately confirm its efficacy and safety, guiding clinicians in making informed decisions that enhance patient care and outcomes.
